# Beyond health aid: would an international equalization scheme for universal health coverage serve the international collective interest?

**DOI:** 10.1186/1744-8603-10-41

**Published:** 2014-05-21

**Authors:** Gorik Ooms, Rachel Hammonds, Attiya Waris, Bart Criel, Wim Van Damme, Alan Whiteside

**Affiliations:** 1 Department of Public Health, Institute of Tropical Medicine, Nationalestraat 155, 2000 Antwerpen, Belgium; 2 School of Public Health, University of the Western Cape, Private Bag X17, Bellville 7535, South Africa; 3 School of Law, University of Nairobi, Parklands Campus, P.O. Box 30197, 00100 Nairobi, Kenya; 4 Balsillie School of International Affairs, Wilfrid Laurier University, 67 Erb Street West, Waterloo, Canada

**Keywords:** Foreign aid, International collective interest, Universal health coverage, Social policy, Equalization, Race to the bottom, Common pool resource

## Abstract

It has been argued that the international community is moving ‘beyond aid’. International co-financing in the international collective interest is expected to replace altruistically motivated foreign aid. The World Health Organization promotes ‘universal health coverage’ as the overarching health goal for the next phase of the Millennium Development Goals. In order to provide a basic level of health care coverage, at least some countries will need foreign aid for decades to come. If international co-financing of global public goods is replacing foreign aid, is universal health coverage a hopeless endeavor? Or would universal health coverage somehow serve the international collective interest?

Using the Sustainable Development Solutions Network proposal to finance universal health coverage as a test case, we examined the hypothesis that national social policies face the threat of a ‘race to the bottom’ due to global economic integration and that this threat could be mitigated through international social protection policies that include international cross-subsidies – a kind of ‘equalization’ at the international level.

The evidence for the race to the bottom theory is inconclusive. We seem to be witnessing a ‘convergence to the middle’. However, the ‘middle’ where ‘convergence’ of national social policies is likely to occur may not be high enough to keep income inequality in check.

The implementation of the international equalization scheme proposed by the Sustainable Development Solutions Network would allow to ensure universal health coverage at a cost of US$55 in low income countries-the minimum cost estimated by the World Health Organization. The domestic efforts expected from low and middle countries are far more substantial than the international co-financing efforts expected from high income countries. This would contribute to ‘convergence’ of national social policies at a higher level. We therefore submit that the proposed international equalization scheme should not be considered as foreign aid, but rather as an international collective effort to protect and promote national social policy in times of global economic integration: thus serving the international collective interest.

## Background

According to Riddell, the principle that underpins foreign aid is simple: "Those who can should help those who are in extreme need" [1]. But Severino and Ray predict the end of foreign aid as we know it: the death of official development assistance (ODA) and its rebirth as global policy financing (GPF) [2]. Sumner and Mallet argue that the future of foreign aid, or ‘Aid 2.0’, will be characterized by co-financing global public goods-and fighting poverty as a global public bad [3]. Glennie proposes ‘international public financing’ instead of foreign aid, and argues that international public financing "should not only be seen as support to other countries, but to the global commons" [4]. With regards to global health, Kaul and Gleicher argue that "[a]s the institution of the state has no full equivalent internationally, international cooperation has to happen voluntarily; and as past experience has shown, voluntary cooperation is more likely to happen when it makes sense for all, that is, if it is based on a clear and fair win-win agreement" [5]. For Kickbusch, "the best is yet to come" for global health, if it "strengthens its political ability to produce global public goods for health" [6]. What all these forecasts have in common is an expectation that ‘helping those who need help’ will no longer be the main engine of foreign aid; the international collective interest will drive international co-financing.

Meanwhile, the World Health Organization (WHO) proposes ‘universal health coverage’ (UHC) as a "single overarching health goal" for the next iteration of the Millennium Development Goals (MDGs) [7], but acknowledges that "[i]n lower-income countries, where prepayment structures may be underdeveloped or inefficient and where health needs are massive, there are many obstacles to raising sufficient funds through prepayment and pooling", and that "[i]t is essential, therefore, that international donors lend their support" [8]. But why would ‘donors’-a misnomer when it comes to co-financing out of collective interest – co-finance UHC in low income countries? The international collective interest of infectious disease control is rather obvious, but it is not self-evident how ensuring "that people have access to *all* the services they need including those relating to [non-communicable diseases], mental health, infectious diseases, reproductive health etc." [7], would serve the international collective interest. The ‘Meeting Global Challenges’ report of the International Task Force on Global Public Goods mentions "preventing the emergence and spread of infectious disease" as a "priority global public good"; it does not mention ‘improving global health’ or ‘reducing global health inequalities’ as a priority global public good [9].

The proposal by the Sustainable Development Solutions Network (SDSN) on health in the post-2015 development agenda expects high income countries to mobilize and allocate the equivalent of 0.1% of their gross domestic product (GDP) to international assistance for health [10]. All countries are expected to "make progress to allocating at least 5% of national GDP as public financing for health (with low- and middle-income countries reducing by at least half the gap between 5% of GDP and current public funding)", domestically^[a]^[10]. This proposal may come across as yet another foreign aid proposal – coming with domestic financing conditions or expectations – but we contend that it has the characteristics of a (modest) international ‘equalization’ scheme that could serve the collective international interest. (Our examination uses the SDSN figures for illustrative purposes, but this does not mean we support all the proposed levels of allocation of GDP to public financing for health).

Equalization is a word used to describe mechanisms that are common to most federal countries and that are designed to ensure that sub-national jurisdictions (like the provinces of Canada or the ‘länder’ of Germany) can – in spite of their fiscal autonomy and differences in economic activity – provide comparable levels of public services [11]. The Canadian Constitution Act, for example, imposes equalization "to ensure that provincial governments have sufficient revenues to provide reasonably comparable levels of public services at reasonably comparable levels of taxation" [12].

The SDSN proposal is somewhat similar to an equalization scheme, in that it expects comparable levels of government revenue raising from all *countries* (instead of provinces), and it would ensure that all countries can provide at least basic levels of public health services. The SDSN proposal would not allow all countries to provide *comparable* levels of public health services, at least not in the short term. Over time, if GDP per capita levels would converge, and if cross-subsidies between countries would increase, an international equalization scheme would allow all countries to provide comparable levels of public health services.

Would an international equalization scheme be possible, without an international government? According to Holst, The European Social Fund and the European Cohesion Fund can be seen as equalization schemes, while the European Commission, which manages these funds, is not a government [13]. Nonetheless, we do not have at the global level an international organization with the powers of the European Commission. But the purpose of this paper is not to explore how an international equalization scheme for universal health coverage could be organized; the purpose is to explore one of the arguments for such a scheme – an argument that has received limited attention in the context of international aid.

Why would high income countries voluntarily enter a international equalization scheme that – at least in the short term – will only cost them financial contributions? Where is the "clear and fair win–win agreement" that Kaul and Gleicher are looking for [5]; where is Kickbusch’s global public good for health [6]? The SDSN emphasizes an expected ‘externality’ of UHC that highlights its global public good value, namely economic growth [9]. Other externalities of UHC have been suggested and examined for their global public good value elsewhere: infectious disease control, demographic control (encouraging the ‘demographic transition’ through improved health care), increasing security and decreasing pressure for migration [14]. They may all be valid and contribute to the political feasibility of the international equalization scheme proposed by the SDSN. In this paper, however, we want to explore a different externality, namely the impact that an international equalization scheme could have on the so-called ‘race to the bottom’.

For this purpose, we explore the double hypothesis that national social policies "face the threat of a ‘race to the bottom’" due to global economic integration, and that this threat can be reversed or mitigated through international social policies [15]. The global equalization scheme as proposed would be, if accepted, an international social policy. If all countries agreed to observe the minimum levels of domestic public health financing proposed by the SDSN, many would have to adapt taxation levels accordingly, and that could mitigate the downward pressure on taxation and social policy levels caused by the quest for competitiveness in a globalized market – or so we will examine.

To be clear, we consider the global equalization scheme proposed by the SDSN first and foremost as a practical implementation of the shared national and international responsibility enshrined in the human right to health [16], and we would support it even if it had no global public good value. But we contend that it would serve the international collective interest, and it should be considered and examined as an international collective effort to protect and promote national social policies, rather than as a new foreign aid proposal.

## Discussion

### An international equalization scheme for universal health coverage: implications for low, middle and high income countries

The SDSN proposes that all countries "make progress to allocating at least 5% of national GDP as public financing for health (with low- and middle-income countries reducing by at least half the gap between 5% of GDP and current public funding)", domestically [10].

To understand the financial implications of this proposal, we need to compare the proposed domestic public financing levels with the present domestic public financing levels. The WHO World Health Statistics 2013 report provides us with estimates of average total health expenditure as percentage of GDP in different income groups of countries, and with estimates of average government expenditure on health as percentage of total health expenditure, both for 2000 and 2010 [17]. For low income countries in particular, there is an additional correction to be made to examine *domestic* public financing levels: external resources for health are reported as percentage of total health expenditure; thus we cannot determine how much international assistance is included in government expenditure and how much international assistance is included in private health expenditure. Table 1 is based on the assumption that international assistance is proportionally allocated to government and private expenditure, and that means that low income countries were allocating, in 2010, the equivalent of 1.5% of GDP to domestic public financing for health.

**Table 1:** Health expenditure in low, middle and high income countries (data for 2010)

**Income group**	**Total expenditure on health as percentage of GDP**	**General government expenditure on health as percentage of total expenditure on health**	***General government expenditure on health as percentage of GDP***	**External resources for health as percentage of total expenditure on health**	***General government expenditure on health form domestic resources, as percentage of GDP***	**General government expenditure on health as percentage of GDP, SDSN proposal**
Low income	5.3	38.5	*2.0*	26.3	*1.5*	3.25
Lower middle income	4.3	36.1	*1.6*	2.5	*1.5*	3.25
Upper middle income	6.0	55.5	*3.3*	0.3	*3.3*	4.15
High income	12.4	61.8	*7.7*	1.1	*7.6*	5.00

Source: World Health Organization, World Health Statistics 2013.

This table shows that the SDSN proposal is very demanding for low income and lower middle income countries: they are expected to increase government expenditure on health from domestic resources from 1.5% of GDP to 3.25% of GP (to halve the gap between 1.5% and 5% of GDP). Upper middle income countries are expected to make additional efforts as well, while high income countries have, on average at least, already reached their target.

But low income countries – and some lower middle income countries – would also benefit from international transfers under the proposed international equalization scheme. To calculate how much they would benefit, we developed a spreadsheet based on data for 2011 from the Global Health Observatory of the WHO [18], and assumed that the equivalent of 0.1% of GDP that high income countries are expected to contribute to international co-financing would be distributed in accordance with needs: the poorest countries would come first. First we assumed that all countries that are not yet allocating the equivalent of 5% of GDP to government expenditure on health would indeed halve the gap between their present spending level and 5% of GDP, than we distributed US$45 billion – the equivalent of 0.1% of GDP of the ‘advanced economies’, according to the International Monetary Fund (IMF) [19] – starting from the poorest countries. Table 2 shows the results.

**Table 2:** Distribution of equalization transfers (data for 2011)

**Country**	**Present per capita government expenditure on health at average exchange rate (US$)**	**General government expenditure on health as percentage of GDP after reduction of external resources**	**Minimum domestic general government expenditure on health as percentage of GDP, SDSN proposal**	**Minimum domestic per capita government expenditure on health, SDSN proposal, at average exchange rate (US$)**	**Gap between minimum domestic per capita expenditure and US$55**	**Population (in thousands of people)**	**Equalization transfers required (in thousands of US$)**
India	18.32	1.19	3.09	47.24	7.76	1,220,000	9,463,809.96
Bangladesh	9.71	1.27	3.14	22.37	32.63	153,000	4,991,904.92
Ethiopia	9.59	1.49	3.25	11.60	43.40	89,393	3,879,572.30
Pakistan	8.02	0.64	2.82	33.37	21.63	176,000	3,806,852.47
Democratic Republic of the Congo	6.66	1.98	3.49	8.06	46.94	63,932	3,001,283.16
United Republic of Tanzania	14.75	1.69	3.35	17.15	37.85	46,355	1,754,549.09
Uganda	11.15	1.81	3.41	15.29	39.71	35,148	1,395,846.96
Myanmar	2.92	0.26	2.63	29.62	25.38	52,351	1,328,480.95
Kenya	14.34	1.09	3.04	24.57	30.43	42,028	1,278,957.14
Afghanistan	8.72	1.25	3.12	18.24	36.76	29,105	1,069,823.02
Mozambique	14.70	0.83	2.92	15.59	39.41	24,581	968,847.64
Nepal	12.98	1.83	3.41	20.72	34.28	27,156	931,027.03
Madagascar	11.98	2.11	3.56	16.59	38.41	21,679	832,587.08
Niger	11.09	2.11	3.56	13.44	41.56	16,511	686,172.11
Malawi	22.71	2.93	3.96	14.63	40.37	15,458	624,066.51
Nigeria	29.19	1.85	3.42	51.20	3.80	164,000	623,569.96
Burkina Faso	18.70	1.92	3.46	19.78	35.22	15,995	563,322.38
Mali	20.25	2.29	3.64	23.85	31.15	14,417	449,024.64
Burundi	7.63	1.51	3.26	8.72	46.28	9,540	441,493.12
Guinea	8.13	1.43	3.21	16.03	38.97	11,162	434,953.54
Cambodia	11.50	1.08	3.04	27.35	27.65	14,606	403,917.74
Sudan	29.40	2.27	3.64	44.89	10.11	36,431	368,314.98
Chad	9.55	0.99	2.99	24.62	30.38	12,080	366,939.02
Rwanda	35.58	3.28	4.14	24.13	30.87	11,144	344,008.31
Côte d’Ivoire	21.14	1.60	3.30	38.68	16.32	19,390	316,452.49
Haiti	25.20	2.45	3.72	27.01	27.99	10,033	280,808.39
Benin	19.55	1.59	3.30	26.47	28.53	9,780	279,012.07
Cameroon	21.23	1.56	3.28	42.78	12.22	21,156	258,549.98
Sierra Leone	12.34	2.72	3.86	14.04	40.96	5,865	240,231.21
Eritrea	6.78	0.39	2.69	14.62	40.38	5,933	239,554.77
Togo	23.45	3.46	4.23	23.70	31.30	6,472	202,574.69
Tajikistan	15.99	1.46	3.23	30.24	24.76	7,815	193,501.05
Liberia	17.34	2.62	3.81	10.74	44.26	4,080	180,582.19
Central African Republic	9.49	1.27	3.14	15.12	39.88	4,436	176,923.50
Viet Nam	38.25	2.66	3.83	53.33	1.67	89,914	150,470.00
Senegal	39.05	3.00	4.00	44.78	10.22	13,331	136,176.99
Yemen	18.45	1.09	3.05	49.27	5.73	23,304	133,454.66
Lao People’s Democratic Republic	18.11	1.04	3.02	40.09	14.91	6,521	97,238.62
Gambia	14.82	1.25	3.12	19.52	35.48	1,735	61,566.39
Guinea-Bissau	9.97	0.92	2.96	17.53	37.47	1,624	60,854.17
Kyrgyzstan	42.52	3.46	4.23	46.44	8.56	5,403	46,269.47
Mauritania	34.95	3.01	4.00	42.87	12.13	3,703	44,920.14
Timor-Leste	33.11	1.78	3.39	30.99	24.01	1,096	26,316.01
Comoros	24.60	1.81	3.40	27.53	27.47	700	19,227.96
Zambia	52.15	2.66	3.83	54.61	0.39	13,634	5,319.01
South Sudan	13.46	0.55	2.78	54.63	0.37	10,381	3,846.39
Sao Tome and Principe	39.02	1.92	3.46	52.55	2.45	183	448.22
							43,163,622.42

Source: World Health Organization, Global Health Observatory data repository (consulted April 2014), SDSN proposal.

All low income and some lower middle income countries would receive international co-financing for UHC; the combination of increased domestic efforts and international co-financing would allow them to spend about $55 per person per year on UHC. All countries not mentioned in Table 2 would be able to spend the same amount or more, from domestic resources only. Would that be sufficient? The Taskforce on Innovative International Financing for Health Systems estimated that, in low income countries, the costs of achieving the current health sector MDGs would be about $50-55 per person per year [20].

Table 1 already illustrated that the effort expected from low and middle income countries is – in percentage of GDP – far more substantial than the effort expected from high income countries. According to our estimates, all low and middle income countries together are expected to increase general government expenditure by about $267 billion, or six times more than the effort expected from high income countries.

**Figure 1:**
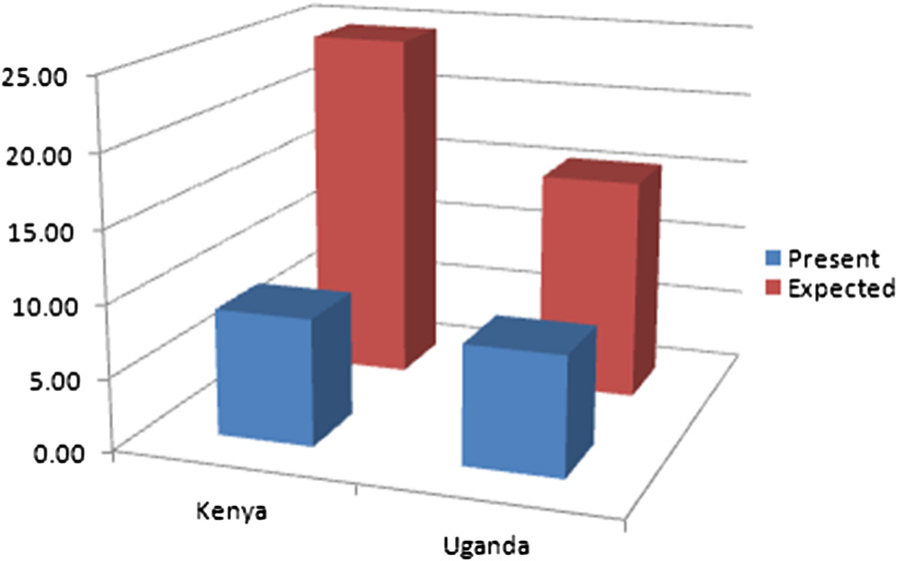
Present and expected public financing of UHC in Kenya and Uganda, per person per year.

**Figure 2:**
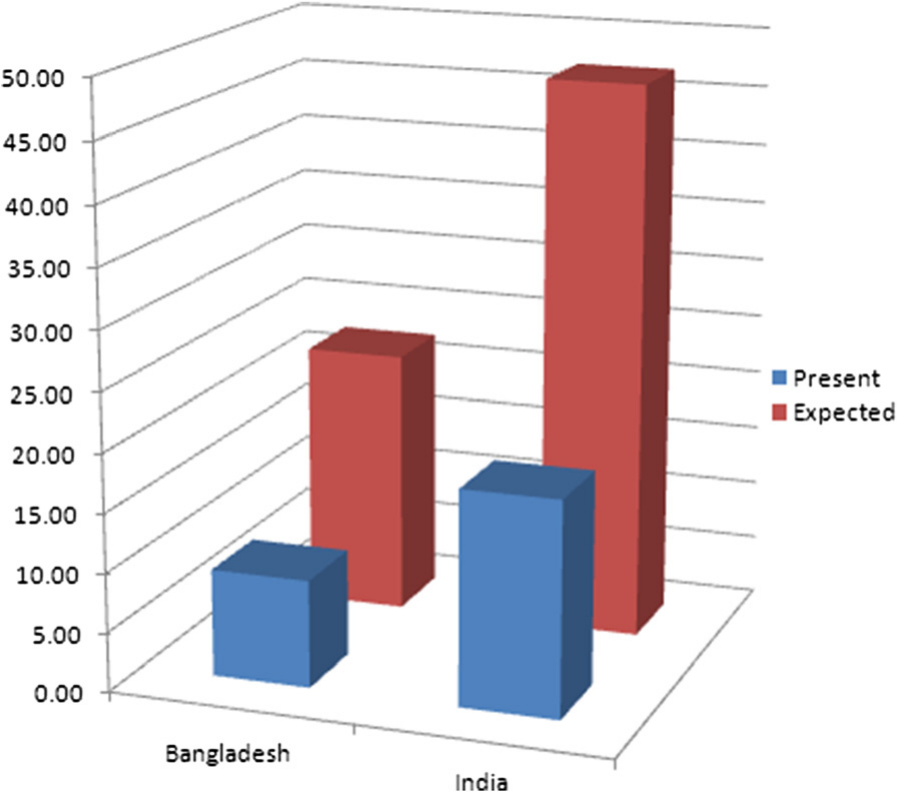
Present and expected public financing of UHC in Bangladesh and India, per person per year.

**Figure 3:**
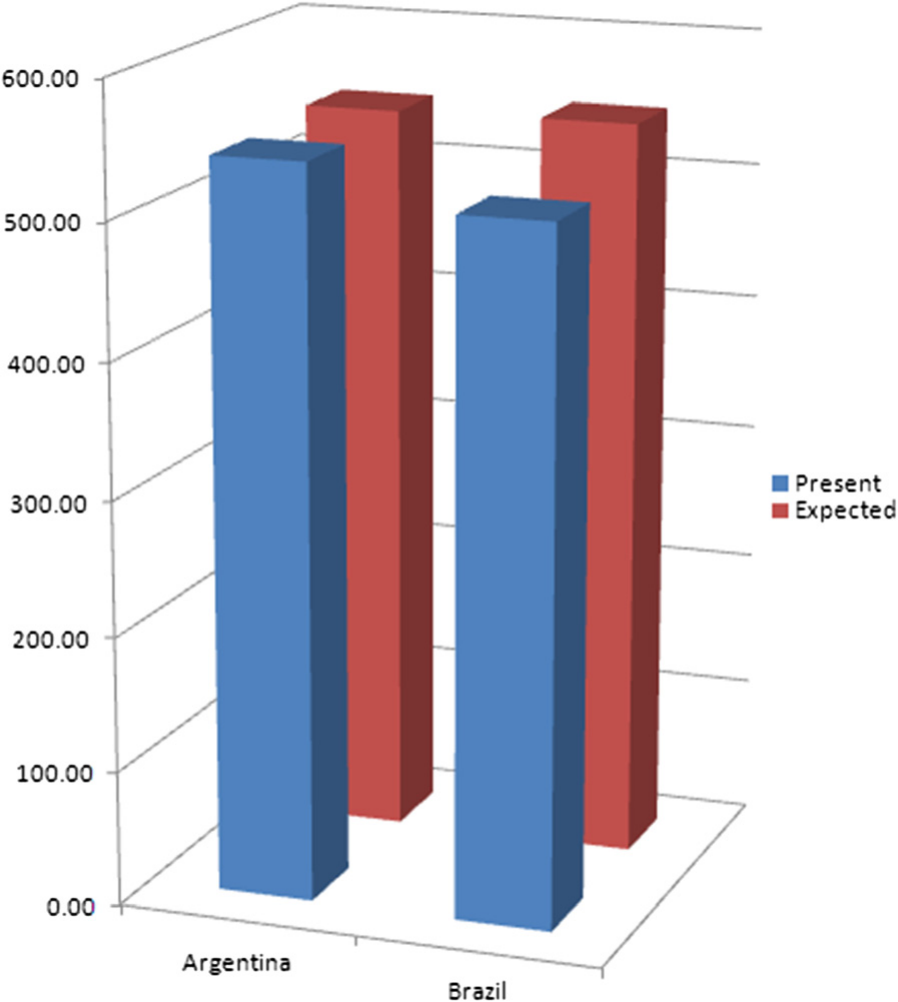
Present and expected public financing of UHC in Argentina and Brazil, per person per year.

Figure 1 illustrates the additional domestic effort expected from Uganda and Kenya, two low income neighboring countries in Africa. Figure 2 illustrates the additional domestic effort expected from Bangladesh and India, two neighboring countries in Asia. Figure 3 illustrates the additional domestic effort expected from Argentina and Brazil, two upper middle income neighboring countries in Latin America.

We used three pairs of neighboring countries with comparable levels of economic development because of the race to the bottom theory, which we will examine in the next section.

### ‘Race to the bottom’, true or false?

In 1997, Rodrik warned against "social disintegration as the price of economic integration" [21]. One consequence of global economic integration is that some factors of the economy, like highly skilled workers and capital, can easily move from countries where they (or their owners) consider the tax burden as detrimental to their profits, to produce similar goods and services in countries where taxation is lower, and to sell these products in the markets of the countries they moved out from. Companies based in countries with a (relatively) higher tax burden are forced to compete with those in countries with a lower tax burden, which find it easier to attract investment and highly skilled workers. Governments of countries with higher tax burdens are therefore encouraged to reduce taxation levels, at times at the expense of national social policy. Conversely, governments wishing to enhance their national social policies may not be doing so, out of fear of becoming less attractive for investment. As Manmohan Singh, then Finance Minister and now Prime Minister of India, explained to Friedman: "In a world in which capital is internationally mobile, you cannot adopt rates of taxation that are far from the rates that prevail in other countries and when labor is mobile you also can’t be out of line with others’ wages" [22].

**Figure 4:**
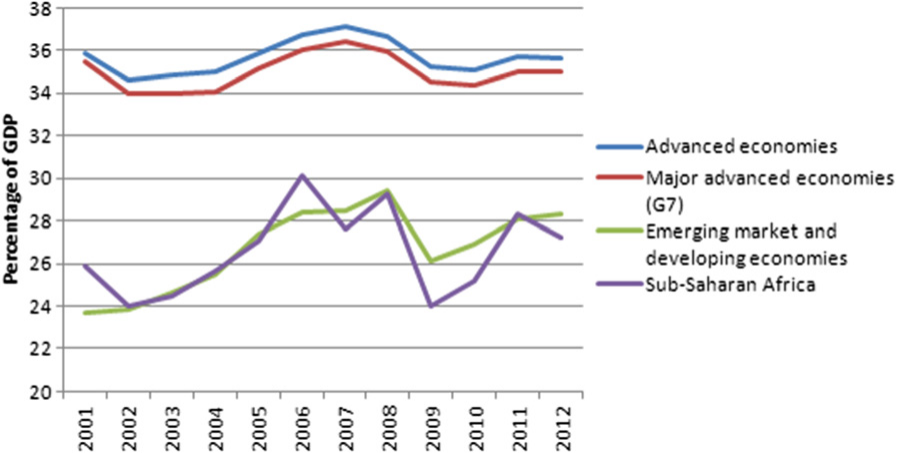
Recent evolution of general government revenue in aggregated groups of countries.

What is the evidence for this ‘race to the bottom’? The World Economic Outlook dataset of the IMF provides information about general government revenue in aggregated groups of countries [19]. As Figure 4 illustrates, the average general government revenue in ‘advanced economies’ (corresponding with the World Bank’s high income economies) seems to decrease very slowly – not a ‘race’ at all – from 35.9% of GDP in 2001 to 35.6% of GDP in 2012: that is a decrease of 0.3% of GDP. That seems may seem negligible, but it is three times the volume of international co-financing of UHC expected by the SDSN. The seven biggest economies or ‘G7’ followed the same path at a similar pace: government revenue decreased from 35.5% of GDP in 2001 to 35% in 2012, a decrease of 0.5% of GDP. But the average general government revenue of ‘emerging market and developing economies’ (corresponding with the World Bank’s low and middle income economies) increased from 23.7% of GDP in 2001 to 28.3% of GDP in 2012. Rather than a race to the bottom, we seem to witness ‘convergence towards the top’.

A closer look at the same dataset – zooming in on the G7 countries – tells a more nuanced story. In France, Italy, and Japan, government revenue increased substantially. In Germany and the UK, it remained more or less stable, but in Canada, and the USA, government revenue decreased substantially. In the USA, representing almost half of the GDP of all G7 countries combined, government revenue decreased from 32.1% of GDP in 2001 to 29% of GDP in 2012: that is a decrease of 3% of GDP. In Canada, the ‘loss’ was even worse: from 45.1% of GDP in 2001 to 41.5% of GDP in 2012, or a 3.6% of GDP decrease.

As it appears, countries with a government revenue level hovering around 50% of GDP (e.g., France) are able to ‘coexist’ within a relatively open trade relationship with countries with a much lower government revenue level hovering around 30% of GDP (e.g., Japan), without facing a massive exodus of investment. Ambitious social policy can also improve competitiveness. Intergenerational social mobility – or "the extent to which individuals move up (or down) the social ladder compared with their parents" – is influenced by many factors, some of which are "heavily affected" by social policy, including "policies that shape access to human capital formation, such as public support for early childhood, primary, secondary and tertiary education, as well as redistributive policies (e.g. tax and transfer schemes) that may reduce or raise financial and other barriers to accessing higher education" [23]. In other words, higher levels of taxation can allow for social policies that encourage intergenerational social mobility. Intergenerational social mobility indicates that more people succeed in developing and using their talents, and therefore one can intuitively expect that in countries with relatively high intergenerational social mobility, in part due to relatively high tax revenue and social policy, the average productivity would be relative high too. That could explain how countries with a government revenue level hovering around 50% of GDP are able to coexist within a relatively open trade relationship with countries with a much lower government revenue level. So, the premise on which the race to the bottom theory is built seems incomplete.

However, at least at some times, some governments decided to cut back on social policy with the intention of increasing the global competitiveness of the companies based in their countries. For example, in the early 1990s, John Major, then Prime Minister of the UK, admitted to "having created a paradise for foreign investors", saying: "Europe can have the social charter. We shall have employment" [24].

**Figure 5:**
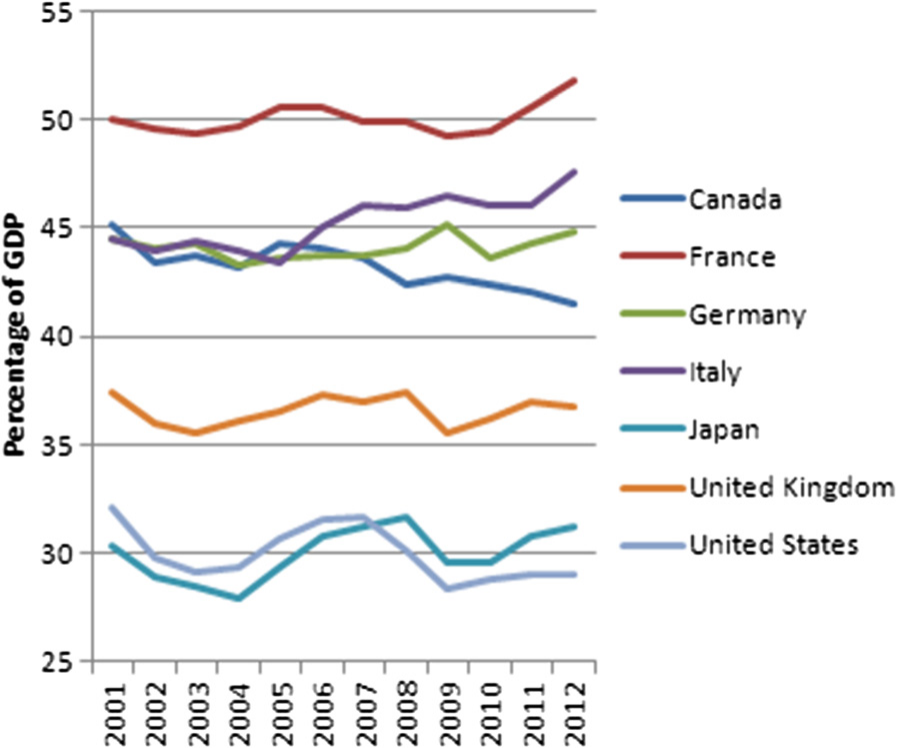
Recent evolution of general government revenue in G7 countries.

According to Boix, there are two very different stories about the historical relationship between global economic integration and national social policy. One is based on the race to the bottom theory, already discussed above: "According to this position, the advanced world will end up adjusting its welfare state downward, forced by the competition of emerging economies" [25]. In the other story, "equally possible and empirically more compelling", or so argues Boix, globalization promotes growth in all open economies, and "as soon as each economy reaches a certain level of prosperity, it expands political rights and democratizes", which "in turn, leads to the creation of a social insurance system" [25]. But he concedes that because of global economic integration, "more mature economies may have to implement some policy adjustments in the short and medium run" [25], and mentions the UK and the USA as examples of countries with "quite flexible labour markets that adjust readily to world prices", which "has resulted, so far, in lower levels of structural unemployment yet higher levels of income inequality", while "long-term unemployment, sustained by labour regulations and unemployment benefits, has rocketed in Europe, especially in those countries in its periphery (such as the Mediterranean basin), which combine weakly competitive industries and very generous welfare systems" [25]. Read in conjunction with Figures 4 and 5, Boix’ comments seem to confirm the argument that there has been a downward erosion, if not a race to the bottom, at least in high income countries.

Still according to Boix, the proponents of the race to the bottom theory are "split into two political camps": the ‘protectionists’, who "would rather stop or even undo the process of international integration", and the ‘federalists’, "by now mostly limited to parts of the academic world and some policy elites", who "defend the construction of global political institutions to unify national regulations (such as labour or environmental standards) in order to counter the effects of excessive capital mobility and inter-state competition" [25]. Based on our support for the SDSN proposal, Boix would probably count us among the ‘federalists’.

But one does not have to be a firm believer in the race to the bottom theory, to argue for international social protection standards. Even if one agrees with Boix, one can still consider that it may be a smart economic option for high income countries to ‘invest’ in international social policy standards, to allow the ‘temporary adjustments’ to be shorter and less invasive, *and* to promote the transition towards higher social protection levels elsewhere. Even if global economic integration were indeed leading to convergence towards the top in the long term, it may be possible and wise to invest in the acceleration of that process.

Furthermore, even if government revenue in low and middle income countries seems to increase faster than the coinciding decrease in high income countries, it may not increase fast enough to mitigate income inequality. According to Milanovic, international income inequality is declining, if measured by comparing average income of countries, weighted for population size [26]. But if measured by comparing household income across borders, the data do "not show any clear trend over the period 1988–2005 for which we have detailed household survey data" [26]. The explanation is that while average income inequality between countries decreases, income inequality within countries increases. Firebaugh comes to similar conclusions and warns that "the transfer of inequality from across nations to within nations is likely to create new problems or exacerbate old ones within nation", and that "[g]rowing income inequality within nations might raise the specter of growing civil unrest and terrorism by nonstate actors at the very time that the effectiveness of national governments is weakened by transnational structures" [27]. If we want declining income inequality between countries to lead to declining income inequality between people, social policy levels in low and middle income countries would have to increase faster than they currently are, while social policy levels in high income countries would have to be stopped from further declining.

### An international equalization scheme for UHC: feasible or wishful thinking?

As discussed above, the international equalization scheme proposed by the SDSN comes with substantial incentives for low income countries like Uganda and Kenya. Uganda would be expected to double its domestic effort – from $8 to $15 per person per year – and Kenya would be expected to almost triple its domestic effort – from $9 to $24 per person per year. But Uganda would receive $40 per person per year from the equalization scheme, while Kenya would receive $31 per person per year from the scheme.

There would be an additional benefit for both Kenya and Uganda. The Government of Kenya may be reluctant to increase government revenue, out of fear that investments will move to neighboring Uganda, for example. And the Government of Uganda may be reluctant to increase government revenue, for exactly the same reason. According to the Tax Justice Network Africa and ActionAid, Kenya, Rwanda, Tanzania and Uganda already are involved in a regional tax competition [28]. If all these countries progress together at a similar pace, none of them would benefit, and none of them would have to fear that their neighbor would benefit to their detriment.

Neither Argentina nor Brazil would receive international cross-subsidies under the international equalization scheme. But Argentina would benefit from Brazil being expected to increase government revenue, while the Government of Brazil may feel more comfortable in doing so – considering expectations from constituencies in Brazil – if it were assured that the Government of Argentina would not use the opportunity to lower its government revenue for the sake of luring away investment.

The real challenge seems to be a problem of ‘free riding behavior’. We can consider the problem of maintaining or increasing competitiveness at the expense of social policy – either decreasing social policy, or not increasing social policy to levels desired by most people – as ‘a tragedy of the commons’. The commons, or ‘common pool resource’ (CPR), is ‘global potential government revenue’. Governments that reduce taxation levels or delay the increase of taxation levels are obviously not trying to get rid of government revenue, they are trying to attract potential government revenue – taxable economic activity – from elsewhere, or trying to prevent it from fleeing. Even if we consider the present evolution of government revenue as a convergence rather than a race to the bottom, there probably is a loss of global potential government revenue due to governments holding each other back. That means that the international community needs a ‘collective choice arrangement’ [29]; countries need to agree on what reasonably comparable levels of taxation and social policy are, which is exactly what the SDNS proposal is proposing (if only for health care). It would only work if all countries participate: Argentina and Brazil need to be sure that Uruguay and Paraguay join the collective choice arrangement, Kenya and Uganda need to be sure that Rwanda and Tanzania join, and so on. But some countries are likely to opt for free riding: every country would benefit from a collective effort of increasing government revenue and social (health) policy, but an individual country could benefit even more from adopting a slightly lower level than the agreed level, thus enjoying the benefits of somewhat higher social policy *and* improved competitiveness.

Let us assume for a while that many countries are interested to join the collective choice arrangement, but some are not – and cannot be forced. Can we solve that? Rodrik, a ‘protectionist’ and a ‘federalist’ at the same time, proposes "two different paths, one appropriate for the short to medium term, and the other for the long term" [30], to reduce the downward pressure from global economic integration on national social policy. His path for the short to medium term is one of reversing global economic integration: countries would be allowed to opt out of international trade agreements and present World Trade Organization (WTO) rules, if that is required to protect their social policies. His path for the long term is "global federalism", in which "politics and jurisdictions expand to match the scope of a truly integrated global economy" [30]. We would argue that his proposal can be used to stimulate convergence towards the top as much as for avoiding downwards erosion, and that the chronology proposed by Rodrik can be reversed – advancing global social integration first, reversing global economic integration second (if still needed).

Allow us to imagine, for the sake of the exercise, that the United Nations General Assembly does not embrace the SDSN proposal, but that the countries of the African, Caribbean and Pacific (ACP) and European Union (EU) partnership do. That partnership was created with the first Lomé Convention of 1975, and it has a Joint Parliamentary Assembly that unites representatives of 78 ACP and 28 EU countries. About 1.4 billion people live in one of these 106 countries – 20% of the world’s population, 50% of the world’s countries. The Joint Parliamentary Assembly has a standing committee on social and environmental affairs, where the SDSN proposal could be discussed and adapted. If we apply the thresholds for domestic efforts and international transfers proposed by the SDSN to the countries of the ACP-EU partnership (excluding all other countries), we find that they could aim together for a minimum level of UHC at almost $50 per person per year in all countries of the partnership. The ‘advanced economies’ of the EU would, together, contribute to equalization transfers at 0.1% of GDP: $16.8 billion per year, as Table 3 illustrates.

**Table 3:** Contributions to equalization transfers ACP-EU (data for 2011)

**Country**	**GDP, current prices, in billions of US$, 2011**	**Contributions to equalization (in thousands of US$)**	**Percentage of GDP of all ‘advanced economies’ of the EU**
Germany	3,631	3,631,435	21.76
France	2,785	2,784,761	16.68
United Kingdom	2,465	2,464,639	14.77
Italy	2,198	2,198,350	13.17
Spain	1,456	1,455,867	8.72
Netherlands	834	833,519	4.99
Sweden	536	536,001	3.21
Belgium	514	513,790	3.08
Austria	416	416,365	2.49
Denmark	334	333,744	2.00
Greece	290	290,153	1.74
Finland	263	262,620	1.57
Portugal	238	238,106	1.43
Ireland	226	226,242	1.36
Czech Republic	216	216,061	1.29
Slovak Republic	96	95,971	0.57
Luxembourg	58	58,063	0.35
Slovenia	50	50,299	0.30
Latvia	28	28,480	0.17
Cyprus	25	25,017	0.15
Estonia	23	22,564	0.14
Malta	9	9,314	0.06
	16,691	16,691,361	100.00

Source: International Monetary Fund, World Economic Outlook Database, April 2014.

On the distribution side, several beneficiaries would ‘disappear’ because they are not ACP members, thus reducing the need substantially. Even so, $55 per person per year would not be possible, but $50 would, almost – it would require $17 billion per year, as Table 4 illustrates.

**Table 4:** Distribution of equalization transfers ACP-EU (data for 2011)

**Country**	**Present per capita government expenditure on health at average exchange rate (US$)**	**General government expenditure on health as a percentage of GDP after reduction of external resources**	**Minimum domestic general government expenditure on health as a percentage of GDP, SDSN proposal**	**Minimum domestic per capita government expenditure on health, SDSN proposal, at average exchange rate (US$)**	**Gap between minimum domestic per capita expenditure and US$50**	**Population (in thousands) total**	**Equalization transfers required**
Ethiopia	9.59	1.49	3.25	11.60	38.40	89,393	3,432,607.30
Democratic Republic of the Congo	6.66	1.98	3.49	8.06	41.94	63,932	2,681,623.16
United Republic of Tanzania	14.75	1.69	3.35	17.15	32.85	46,355	1,522,774.09
Uganda	11.15	1.81	3.41	15.29	34.71	35,148	1,220,106.96
Kenya	14.34	1.09	3.04	24.57	25.43	42,028	1,068,817.14
Mozambique	14.70	0.83	2.92	15.59	34.41	24,581	845,942.64
Madagascar	11.98	2.11	3.56	16.59	33.41	21,679	724,192.08
Niger	11.09	2.11	3.56	13.44	36.56	16,511	603,617.11
Malawi	22.71	2.93	3.96	14.63	35.37	15,458	546,776.51
Burkina Faso	18.70	1.92	3.46	19.78	30.22	15,995	483,347.38
Mali	20.25	2.29	3.64	23.85	26.15	14,417	376,939.64
Burundi	7.63	1.51	3.26	8.72	41.28	9,540	393,793.12
Guinea	8.13	1.43	3.21	16.03	33.97	11,162	379,143.54
Sudan	29.40	2.27	3.64	44.89	5.11	36,431	186,159.98
Chad	9.55	0.99	2.99	24.62	25.38	12,080	306,539.02
Rwanda	35.58	3.28	4.14	24.13	25.87	11,144	288,288.31
Côte d’Ivoire	21.14	1.60	3.30	38.68	11.32	19,390	219,502.49
Haiti	25.20	2.45	3.72	27.01	22.99	10,033	230,643.39
Benin	19.55	1.59	3.30	26.47	23.53	9,780	230,112.07
Cameroon	21.23	1.56	3.28	42.78	7.22	21,156	152,769.98
Sierra Leone	12.34	2.72	3.86	14.04	35.96	5,865	210,906.21
Eritrea	6.78	0.39	2.69	14.62	35.38	5,933	209,889.77
Togo	23.45	3.46	4.23	23.70	26.30	6,472	170,214.69
Liberia	17.34	2.62	3.81	10.74	39.26	4,080	160,182.19
Central African Republic	9.49	1.27	3.14	15.12	34.88	4,436	154,743.50
Senegal	39.05	3.00	4.00	44.78	5.22	13,331	69,521.99
Gambia	14.82	1.25	3.12	19.52	30.48	1,735	52,891.39
Guinea-Bissau	9.97	0.92	2.96	17.53	32.47	1,624	52,734.17
Mauritania	34.95	3.01	4.00	42.87	7.13	3,703	26,405.14
Comoros	24.60	1.81	3.40	27.53	22.47	700	15,727.96
							17,016,912.94

Source: World Health Organization, Global Health Observatory data repository (consulted April 2014), SDSN proposal.

If the 106 countries of the ACP-EU partnership were to agree on an ACP-EU equalization scheme for health, along the lines of the SDSN proposal, they would have enough influence within the WTO to negotiate less preferential treatment for non-adhering countries. This presupposes, however, that WTO members that are not ACP-EU countries are also allowed to adhere – they would have the choice between adhering and enjoying preferential trade status, or not adhering and not enjoying the preferential trade status. Thus global economic integration would be reversed, but only for countries rejecting the collective choice arrangement. The collective choice arrangement would not be imposed upon sovereign states, but it would come with an additional benefit. If such a solution can be found for international standards of intellectual property protection – the WTO Agreement on Trade Related Aspects of Intellectual Property Rights (TRIPS) – why not for international standards of social policy?

Obviously, more research is needed to be able to predict the potential impact of the SDSN proposal on national social policy, government revenue, investment and trade, but we submit – for further debate and research – that the proposed international equalization scheme should not be considered as a foreign aid proposal, but rather as an international collective effort to protect and promote national social policy in times of global economic integration.

## Summary

The interaction between taxation, social policy, and economic growth is not an exact science. Nonetheless, efforts are being made to examine how global economic integration affects the space for national social policy. Most often, however, these do not include models or scenarios considering the option of an international social protection regime.

In 1994, de Swaan considered "a transnational social system in which rich countries collectively pay for benefits to poor people in poor countries" and argued "the question of its feasibility and efficacy merits serious discussion among students of social policy which, to my knowledge, it has so far not received" [31]. But de Swaan was skeptical himself, commenting that "[t]he rich were ready to shoulder the care for the poor only if they believed they could pacify those who might otherwise constitute a threat to them or if the continued presence of the poor in their midst held some opportunities for them" [31]. In the 21st Century, ‘in their midst’ should no longer be considered in geographical terms but in economic terms: if the common people of the wealthier countries want the benefits of global economic integration without losing the benefits of social policy, they will have to support social integration beyond borders.

Although we believe that ethical arguments alone should be sufficient to consider a global social protection regime, what we propose here, for debate, is the hypothesis that global social integration would both support social policy in low income countries, and may help avoid the gradual erosion of social protection in high income countries. The additional cost for high income countries would, in most cases, be limited to their contributions to international transfers as most already expend domestically the minimum required under this proposal.

Finally, we can anticipate the critique that a global social protection regime for health alone will not produce the benefits in terms of mitigating a race to the bottom or promoting convergence towards the top. While we agree that a global social protection regime will only reveal its fullest global public good potential if it aims for a comprehensive package of social protection, we believe that in the area of health, some progress has been made that does not (yet) have its equivalent in other areas of social protection, like unemployment allowances, child benefits or retirement pensions.

Given the orders of magnitude of government revenue at stake due to global economic integration, we conclude that the global equalization scheme for UHC proposed by the SDSN should not be examined as a new form of foreign aid, but rather as an international collective effort to protect and promote national social policy in times of global economic integration.

### Endnote

^a^WHO data on ‘general government expenditure on health’ include mandatory social protection contributions. It is not entirely clear if SDSN includes these contributions as well, when it proposes that all states allocate the equivalent of 5% of national GDP as public financing for health, but we think it does: when it discusses financing in these words: "This has been attributed to the compulsory nature of general taxation and other government revenue sources (e.g. royalties on the exploitation of natural resources) and social health insurance contributions" [10]. We therefore used WHO data on ‘general government expenditure on health’ to assess how far states are removed from the SDSN target for domestic public financing, after excluding external resources proportionally.

## Abbreviations

ACP: African, Caribbean and Pacific; CPR: Common Pool Resource; EU: European Union; GDP: Gross domestic product; GPF: Global policy financing; IMF: International Monetary Fund; MDG: Millennium Development Goal; ODA: Official development assistance; OECD: Organization for Economic Co-operation and Development; SDSN: Sustainable Development Solutions Network; UHC: Universal health coverage; WEO: World Economic Outlook; WHO: World Health Organization; WTO: World Trade Organization.

## Competing interests

The authors declare that they have no competing interests.

## Authors’ contributions

The conceptualization of this paper started at a workshop on global social protection, organized by the Hélène De Beir Foundation and medico international in Berlin in May 2012, by AlW, AtW, GO and RH. GO wrote a first draft, revised by BC and WVD. All authors contributed to further revisions and endorse the final version.

## Authors’ information

GO is a human rights lawyer, researcher at the Public Health Department of the Institute of Tropical Medicine in Antwerp and the Law and Development Research Group at the University of Antwerp, and adjunct professor of law at Georgetown University in Washington DC. Although he believes that the human right to health ought to be a sufficient basis for more and better international assistance for health, the practice of international assistance reveals the importance of clarifying the international collective interest.

RH is a researcher at the Public Health Department of the Institute of Tropical Medicine in Antwerp, a PhD candidate at the VUB University in Brussels, and a member of the Law and Development Research Group at the Law Faculty of the University of Antwerp. Her research focuses on the intersection between human rights, health and development. She is a New York State licensed attorney and has worked at the FXB Center for Health and Human rights at the Harvard School of Public Health.

AtW is a senior lecturer at the Department of Commercial Law of the University of Nairobi and a visiting lecturer at the National University of Rwanda. She is a member of the OECD Informal Task-force on Tax and Development. She holds a PhD in Tax Law and Development from Lancaster University and holds two Masters of Laws, one in Human Rights and Democratization in Africa from the University of Pretoria and another in Business and Commercial Law from the University of London.

BC is a medical doctor by training. He is currently associate professor at the Public Health Department of the Institute of Tropical Medicine in Antwerp where he is heading the health financing unit. He obtained his PhD in the field of district-based health insurance in sub-Saharan Africa. He was chair of the Public Centre of Social Welfare in his home municipality in the period 2001–2008. His fields of interest are the organization and management of local health care delivery systems and design and evaluation of social health protection programs with a geographical focus on sub-Sahara Africa and India.

WVD is a medical doctor and professor in public health & health policy at the Institute of Tropical Medicine, Antwerp and at the School of Public Health, University of Western Cape, South Africa, where he holds the SARChI chair in Health Systems and Social Change. He is lecturer at the Paris School of International Affairs in Paris; and at VUB University in Brussels. His main research interests are related to health policy and health systems strengthening in fast changing societies. He is vice-chair of the Technical Evaluation Reference Group of the Global Fund to fight AIDS, Tuberculosis and Malaria.

AlW is CIGI Chair in Global Health Policy at Balsillie School of International Affairs at Wilfrid Laurier University, Waterloo. He has been researching HIV and AIDS since 1987. He was a Commissioner on the Commission on HIV/AIDS and Governance in Africa between 2003 and 2006. He has a commitment to health and development, with Swaziland and Southern Africa as a central concern.

## References

[B1] Riddell RC Does Foreign Aid Really Work? 2007 Oxford: Oxford University Press

[B2] Severino JM Ray O The End of ODA: Death and Rebirth of a Global Public Policy 2009 Washington, DC: Center for Global Development http://www.cgdev.org/sites/default/files/1421419_file_End_of_ODA_FINAL.pdf

[B3] Sumner A Mallett R The Future of Foreign Aid. Development Cooperation and the New Geography of Global Poverty 2013 Basingstoke: Palgrave MacMillan

[B4] Glennie J Sumner A A Manifesto for International Public Finance in the 21st Century The Donors’ Dilemma: Emergence, Convergence and the Future of Aid 2014 London: Global Policy http://www.globalpolicyjournal.com/blog/13/03/2014/donors%E2%80%99-dilemma-manifesto-international-public-finance-21st-century

[B5] Kaul I Gleicher D Governing Global Health: Is Europe Ready? 2011 London: Global Health Europe http://www.globalhealth.ie/uploads/files/GHE_ResearchSeries_GlobalPublicGoods.pdf

[B6] Kickbusch I A game change in global health: the best is yet to come Public Health Rev 2013 35 1 1 20 e-publication ahead of print 10.1007/BF03391687 PMC7099312 32226195

[B7] World Health Organization Positioning Health in the Post-2015 Development Agenda 2012 Geneva: World Health Organization http://www.worldwewant2015.org/bitcache/7c4f4f265f3d2dfdfed54c06afee939039865522?vid=302852&disposition=attachment&op=download

[B8] World Health Organization The World Health Report: Health Systems Financing: The Path to Universal Coverage 2010 Geneva: World Health Organization http://www.who.int/whr/2010/en/index.html 10.2471/BLT.10.078741 PMC2878164 20539847

[B9] International Task Force on Global Public Goods Meeting Global Challenges: International Cooperation in the National Interest. Report of the International Task Force on Global Public Goods 2006 Washington DC: Communications Development Incorporated http://home.kku.ac.th/petmas/Global%20Public%20Goods%20booklet.pdf

[B10] Sustainable Solutions Development Network Health in the Framework of Sustainable Development 2014 New York: Sustainable Solutions Development Network http://unsdsn.org/wp-content/uploads/2014/02/Health-For-All-Report.pdf

[B11] Stark KJ Rich States, Poor States: assessing the design and effect of a U.S. Fiscal Equalization Regime NY Univ Tax Law Rev 2010 63 4 957 1008

[B12] Canada Constitution Act, 1982 http://www.solon.org/Constitutions/Canada/English/ca_1982.html

[B13] Holst J Holst J Implementing the Solidarity Principle Through Financial Equalisation Global Social Protection Scheme: Moving from Charity to Solidarity 2012 Merelbeke: Hélène De Beir Foundation; Frankfurt: medico international

[B14] Ooms G Hammonds R Van Damme W The International Political Economy of Global Universal Health Coverage Background Paper for the Global Symposium of Health Systems Research, Montreux, November 2010 http://healthsystemsresearch.org/hsr2010/images/stories/3international_policy_economy.pdf

[B15] Pestieau P Cantillon B, Marx I Globalisation and Redistribution International Cooperation in Social Security. How to Cope With Globalisation 2005 Antwerpen: Intersentia

[B16] Ooms G Hammonds R Taking up Daniels’ challenge: the case for global health justice Health Hum Rights 2010 12 1 29 46 20930252

[B17] World Health Organization World Health Statistics 2013 2013 Geneva: World Health Organization http://apps.who.int/iris/bitstream/10665/81965/1/9789241564588_eng.pdf

[B18] World Health Organization Global Health Observatory: Data Repositoryhttp://www.who.int/gho/database/en/

[B19] International Monetary Fund World Economic Outlook, April 2014 2014 Washington, DC: International Monetary Fund http://www.imf.org/external/pubs/ft/weo/2014/01/weodata/index.aspx

[B20] Taskforce on Innovative International Financing for Health Systems More Money for Health, and More Health for the Money 2009 Geneva: World Health Organization http://www.internationalhealthpartnership.net/fileadmin/uploads/ihp/Documents/Results___Evidence/HAE__results___lessons/Taskforce_report_EN.2009.pdf

[B21] Rodrik D Has Globalization Gone Too Far? 1997 Washington, DC: Institute for International Economics

[B22] Friedman T The Golden Straightjacket The Lexus and the Olive Tree 2000 New York: Anchor Books

[B23] Organization for Economic Co-operation and Development Going for Growth 2010 2010 Paris: Organization for Economic Co-operation and Development

[B24] Leibfried S Pierson P Leibfried S, Pierson P Semisovereign Welfare States: Social Policy in a Multitiered Europe European Social Policy: Between Fragmentation and Integration 1995 Washington, DC: Brookings

[B25] Boix C Bacchetta M, Jansen M Redistribution Policies in a Globalized World Making Globalization Socially Sustainable 2011 Geneva: International Labour Organization and World Trade Organization

[B26] Milanovic B Global inequality recalculated and updated: the effect of new PPP estimates on global inequality and 2005 estimates J Econ Inequal 2012 10 1 1 18 10.1007/s10888-010-9155-y

[B27] Firebaugh G The new Geography of Global Income Inequality 2003 Cambridge: Harvard University Press

[B28] Tax Justice Network Africa, ActionAid International Tax Competition in East Africa: A Race to the Bottom? 2012 Nairobi: Tax Justice Network Africa; Johannesburg: ActionAid International http://www.actionaid.org/sites/files/actionaid/eac_report.pdf

[B29] Cox M Arnold G Villamayor Tomás S A review of design principles for community-based natural resource management Ecol Soc 2010 15 4 38

[B30] Rodrik D One Economics, Many Recipes: Globalization, Institutions, and Economic Growth 2007 Princeton and Oxford: Princeton University Press

[B31] De Swaan A De Swaan A Perspectives for Transnational Social Policy in Europe Social Policy Beyond Borders: The Social Question in International Perspective 1994 Amsterdam: Amsterdam University Press

